# Marine turtles are only minimally sexually size dimorphic, a pattern that is distinct from most nonmarine aquatic turtles

**DOI:** 10.1002/ece3.8963

**Published:** 2022-06-02

**Authors:** Christine Figgener, Joseph Bernardo, Pamela T. Plotkin

**Affiliations:** ^1^ 14736 Marine Biology Interdisciplinary Program Texas A&M University College Station Texas USA; ^2^ 14736 Department of Biology Texas A&M University College Station Texas USA; ^3^ 14736 Department of Oceanography Texas A&M University College Station Texas USA; ^4^ Costa Rican Alliance for Sea Turtle Conservation & Science (COASTS) Gandoca Costa Rica; ^5^ 14736 Program in Ecology and Evolutionary Biology Texas A&M University College Station Texas USA; ^6^ 14736 Texas Sea Grant Texas A&M University College Station Texas USA

**Keywords:** body size, cheloniidae, dermochelyidae, sexual dimorphism index, testudines

## Abstract

Turtles have been prominent subjects of sexual size dimorphism (SSD) analyses due to their compact taxonomy, mating systems, and habitat diversity. In prior studies, marine turtles were grouped with fully aquatic non‐marine turtles (NMATs). This is interesting because it is well‐established that the marine environment imposes a distinct selective milieu on body form of vagile vertebrates, driven by convergent adaptations for energy‐efficient propulsion and drag reduction. We generated a comprehensive database of adult marine turtle body sizes (38,569 observations across all species), which we then used to evaluate the magnitude of SSD in marine turtles and how it compares to SSD in NMAT. We find that marine turtles are only minimally sexually size dimorphic, whereas NMAT typically exhibit female‐biased SSD. We argue that the reason for this difference is the sustained long‐distance swimming that characterizes marine turtle ecology, which entails significant energetic costs incurred by both sexes. Hence, the ability of either sex to allocate proportionately more to growth than the other is likely constrained, meaning that sexual differences in growth and resultant body size are not possible. Consequently, grouping marine turtles with NMAT dilutes the statistical signature of different kinds of selection on SSD and should be avoided in future studies.

## Graphical abstract

Marine turtles are only minimally sexual size dimorphic, whereas nonmarine aquatic turtles typically exhibit female‐biased sexual size dimorphism. The reason for this difference is likely the sustained long‐distance swimming that characterizes marine turtle ecology, which entails significant energetic costs incurred by both sexes. Hence, the ability of either sex to allocate proportionately more to growth than the other is likely constrained and marine turtles should not be lumped with nonmarine aquatic turtles in future comparative studies of the different kinds of selections causing sexual size dimorphism.

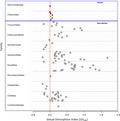

## 1 BACKGROUND

Sexual size dimorphism (SSD) is a phenomenon that has received a great deal of theoretical and empirical attention, as it is thought to reflect variation in sex‐specific selection due to ecological performance, and fitness effects arising from fecundity selection, mating systems, and differences in timing of attainment of sexual maturity between sexes (Fairbairn, 1997; Fairbairn et al., 2007; Lovich et al., 2014; Shine, 1989, 1990). Turtles (Order Testudines) have been prominent model systems for comparative analyses aimed at understanding the causes of SSD, owing to the diversity of their mating systems and habitats (freshwater, terrestrial, marine) they occupy, as well as due to the wide availability of data on body size of many species (Agha et al., 2018; Berry & Shine, 1980; Ceballos et al., 2013; Gibbons & Lovich, 1990; Gosnell et al., 2009; Halámková et al., 2013; Regis & Meik, 2017). All of these analyses assign turtle species to different habitat types (aquatic, semi‐aquatic, terrestrial, etc.), but with varying degrees of detail. In this study, we are reconsidering the practise in prior comparative analyses of grouping marine turtles with other fully aquatic non‐marine turtles (NMAT), or ignoring them all together. Thus, we did not include semi‐aquatic turtles, but only strictly fully aquatic turtles, because prior studies have treated semi‐aquatic species separately (Agha et al., 2018; Halámková et al., 2013).

The seven species of marine turtles comprise a monophyletic lineage (superfamily Chelonioidea) containing two families (Cheloniidae, Dermochelyidae) (reviewed in Figgener et al., 2019). Both extant and extinct marine turtles are well‐known to exhibit striking adaptations to the marine environment including forelimbs highly modified into flippers with concomitant neuromuscular repatterning, and streamlining of body form as is seen in other highly vagile marine vertebrates (Fish, 1993; Kelley & Pyenson, 2015; Pyenson et al., 2014). Three observations pertaining to the marine turtle data that have been used in prior analyses of turtle SSD prompted this study. The first observation is that most reviews do not include data for all seven species (two to five species have been included) although data exist for all seven species in the literature. Second, most studies include species’ mean values that are often based on a single population ignoring a large amount of literature data on body sizes in different populations. Further, some studies report values whose origin in the primary literature is unclear (Table S1). Because most marine turtles occupy far more expansive geographic ranges (Figgener et al., 2019) than any other turtle species including both temperate and tropical regions (Buhlmann et al., 2009), intraspecific diversity in body size may influence overall conclusions about SSD in marine turtles. The third observation is that all the prior analyses cited above are consistent in grouping marine turtles with other fully aquatic turtles despite their well‐known distinct morphology and ecology, which includes long‐distance, often trans‐oceanic migrations (Godley et al., 2008; Hays & Hawkes, 2018; Plotkin, 2003, 2010).

In this paper, we critically examine these observations. First, we address the incompleteness of SSD data for marine turtles in previous studies by assembling the most comprehensive dataset to date on body size of all seven marine turtle species, including estimates from multiple populations within each species. We then analyzed these data to describe quantitatively intraspecific and interspecific patterns in marine turtle SSD. Finally, we compared these new estimates of marine turtle SSD to data from other NMAT to consider an alternative hypothesis to those previously advanced to explain patterns of SSD in fully aquatic turtles (Agha et al., 2018; Berry & Shine, 1980; Ceballos et al., 2013; Gibbons & Lovich, 1990; Gosnell et al., 2009; Halámková et al., 2013), specifically, that the marine environment imposes a distinct selective milieu upon swimming performance as has been well‐established for other large, actively swimming marine vertebrates, and that this should influence the pattern of SSD in marine turtles in contrast to patterns observed in NMAT which do not exhibit lifelong, sustained swimming. To do this, we advance a morphology‐performance‐fitness argument sensu Darwin (1859) or Arnold (1983). With that, we also tested the hypothesis that the previous grouping of marine turtles with other fully aquatic turtles in comparative analyses of SSD is justified or whether it might be advantageous to include marine turtles and NMAT as two separate groups in future comparative analyses.

## 2 METHODS

To test our hypothesis, we reviewed all data on adult marine turtle body size reported and used in prior analyses of turtle SSD to validate their accuracy and to identify any omissions. As part of this process, we re‐examined all the primary sources reported in these studies (summarized in Table S1). Therefore, we generated a new, comprehensive dataset (Table S2) of sex‐specific body sizes (carapace length, CL) of adult marine turtles in which data for both sexes were reported from the same population using data from primary sources.

### 2.1 Methods for compiling dataset of marine turtle body size

To assemble a dataset specifically for marine turtle body size, we started by examining the marine turtle data used in seven comprehensive reviews and analyses aimed at understanding patterns of SSD in turtles (Agha et al., 2018; Berry & Shine, 1980; Ceballos et al., 2013; Gibbons & Lovich, 1990; Gosnell et al., 2009; Halámková et al., 2013; Regis & Meik, 2017). A cursory examination of the datasets used in these papers indicated that reviews were not exhaustive, and not all seven species of marine turtles were included. Further, many datasets simply copied previous data compilations, thus propagating these omissions, as well as certain inaccuracies, even if subsequent studies included new data. Additionally, previous reviews often only included values for a single population and representative of a species. Consequently, we undertook a comprehensive examination of the primary literature to check the accuracy of the used data. To do this, we examined each primary literature source cited in the prior reviews, and we quality checked each data point. A summary of all the primary literature we examined and how it relates to the accuracy and completeness of the data reported in the prior reviews is detailed in Table S1.

Next, we added new data. We conducted a literature search using Google Scholar, SCOPUS and the literature database on SeaTurtle.org to identify any additional primary literature reporting body size data for marine turtles. We included peer‐reviewed studies, student theses, and reports that reported body size data for both sexes within a species and population. We only accepted values when it was clear that they were based on sexually mature adults because data was collected in mating areas adjacent to nesting beaches and/or the size ranges of the sexes were well above the minimum size for the attainment of sexual maturity in that species. We tabulated carapace length means, standard error (SE), and sample sizes. We accepted means from studies of any species that reported the origin of samples, sample size, and either SD or SE within one nesting population or foraging area. We also accepted values from studies for which we could compute means and SE from either original supplementary datasets if available, or from datasets we generated by extracting values from published figures using PlotDigitizer 2.6.88. If multiple estimates (different populations) existed for the same species, we accepted all of them. The resulting dataset (Table S2) included credible estimates from 36 different populations comprising all seven species of marine turtles with most represented by more than one population.

### 2.2 Comprehensive compilation of body size data of marine and NMAT

We generated a dataset for NMAT turtles body size data by combining the information from the two reviews that included data for the largest number of turtle species and because they specifically coded the aquatic species (Agha et al., 2018; Regis & Meik, 2017). Due to taxonomic changes, sometimes data appeared to come from two different species when in fact there were two names that applied to the same species (e.g., an older name and a newer name). Therefore, we reconciled the species’ names using the Annotated Checklist "Turtle Species of the World" (Turtle Taxonomy Working Group et al., 2021). We did not include any of the data for marine turtles from these papers, but rather added our new estimates of species mean values that we calculated from the data in Table S2. We coded each species as marine (M) or nonmarine fully aquatic (A). Our final turtle body size database contains data for 94 fully aquatic species (seven marine and 87 nonmarine aquatic), which is about 50% of all aquatic turtles (Turtle Taxonomy Working Group et al., 2021). Species diversity across the nine NMAT families varies widely, ranging from one (in two monotypic families) to 96 in the Geoemydidae (as of 2021 per Turtle Taxonomy Working Group et al. (2021)). Not all of which are fully aquatic. This unevenness is reflected in our dataset: we tabulated estimates for 87 species overall, representing 10%–100% of the species within each family (Figure 2b and Table S5). The dataset is available in Table S3.

### 2.3 Evaluation of SSD in marine turtles

**FIGURE 1:**
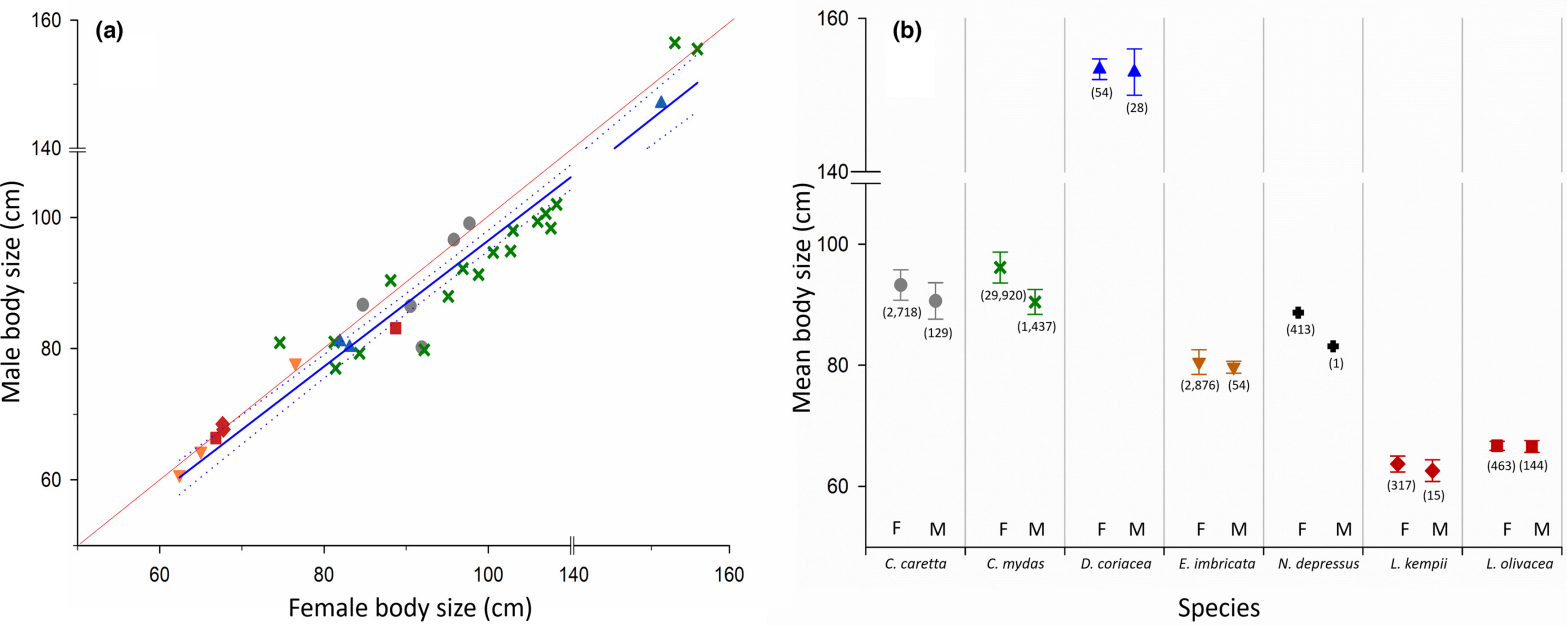
Sex‐specific body size data for seven species of marine turtles based on a new, comprehensive literature review (Table S2). (a) Male versus female carapace length for 36 populations of all seven species of marine turtles: green (green cross: *Chelonia mydas*), loggerhead (dark grey circle: *Caretta caretta*), Kemp's ridley (red diamond: *Lepidochelys kempii*), olive ridley (red square: *Lepidochelys olivacea*), hawksbill (orange inverted triangle: *Eretmochelys imbricata*), flatback (black cross: *Natator depressus*) and leatherback (blue triangle: *Dermochelys coriacea*). The red line is the 1:1 line, representing the null hypothesis of no SSD. The blue line is the regression (male CL = 0.38804 + 0.9611 (female CL); adj *r* ^2^ = 0.96, *p *< .0001), and the dotted lines are the 95% confidence intervals. See text for statistical details. (b) Mean adult body size of the seven species of marine turtles with symbols as in Figure 1a. Number in parentheses is the total sample size across all studies, and error bars indicate 1 Standard Error (SE)

We analyzed this new dataset to test the hypothesis that marine turtles exhibit significant SSD. First, to gain an overview of species differences as well as intraspecific variation, we computed sex‐specific mean values for each population and species using only curved carapace length data, with the exception of *Lepidochelys kempii*, where only straight carapace length data was available. Then we plotted male versus female size for each population and computed a regression of males versus females (Ranta et al., 1994). The null hypothesis, in this case, is that males and females for a given species do not differ in size, which implies a slope of one and an intercept of zero. This null hypothesis thus differs from the standard null in regression analyses that both the slope and intercept are zero. Thus, we used a customized code in R (R Development Core Team, 2018) to test this null hypothesis (see Supporting Information for R Code). Because the data were unbalanced with respect to the number of populations per species (Figure 1a) and species per NMAT family, we repeated the analysis using only mean values for each species and only mean values for each NMAT family. In all three models, each component of the null hypothesis (slope, intercept) was evaluated using a one‐sample, two‐tailed *t*‐test (see Supporting Information for R Code).

### 2.4 Comparison of SSD of marine to NMAT

Data for NMAT were derived from published summaries in two recent analyses of sexual dimorphism in turtles (Agha et al., 2018; Regis & Meik, 2017). Only sex‐specific mean body size data were available in these studies. We aggregated data from the two most comprehensive studies and updated the taxonomic assignments of the studied populations. Additional details of this process and the resultant dataset (Tables S3 and S4) are provided in the Supporting Information.

We used two approaches to test the hypothesis that marine and NMAT should be considered as a single group (aquatic turtles) in analyses of SSD, as has been assumed in previous analyses of turtle SSD (Agha et al., 2018; Berry & Shine, 1980; Ceballos et al., 2013; Gosnell et al., 2009). First, we plotted male versus female mean body size for each species of both nonmarine and marine turtles and fit separate regressions for the two groups. We then conducted an ANOVA in which we included habitat (marine vs nonmarine) as a classification variable. Because there is a correlation between habitat and overall body size (average sizes of marine turtles are far greater than those of NMAT), we might wrongly ascribe to “habitat” a difference driven simply by average size. Therefore, we also included an interaction term between female CL and habitat to account for this association. All regressions and ANOVAs were computed with JMP 13 (JMP^®^, Version 2016. SAS Institute Inc., Cary, NC, 1989–2021).

Second, we computed the Lovich‐Gibbons Sexual Dimorphism Index (SDI_LG_) (Lovich & Gibbons, 1992) for all species, which had two separate equations for female‐ and male‐biased SSD. We are following the suggestion by Fairbairn (1997), limiting the SDI to one equation:

$${\text{SDI}}_{\text{LG}}=\frac{\text{mean size of largest sex}}{\text{mean size of smallest sex}}‐1$$

with female‐biased SSD arbitrarily defined by positive values, 0 indicating no SSD, and male‐biased SSD arbitrarily defined by negative values. We not only plotted these for visual comparison, but we also computed the distributional properties of the SDI_LG_ for each group. We then conducted an ANOVA in which we included habitat (marine vs. nonmarine) as a classification variable. All model effects are interpreted using the Type III Sums of Squares.

## 3 RESULTS

### 3.1 SSD in marine turtles

We obtained reliable body size data for 36 populations nesting and mating at geographically distinct locations (as defined by the original authors) representing all seven species of marine turtles, with a total sample size of 38,569 individuals (36,761 females; 1808 males), the most comprehensive dataset of marine turtle body size to date (Table S2).

**FIGURE 2:**
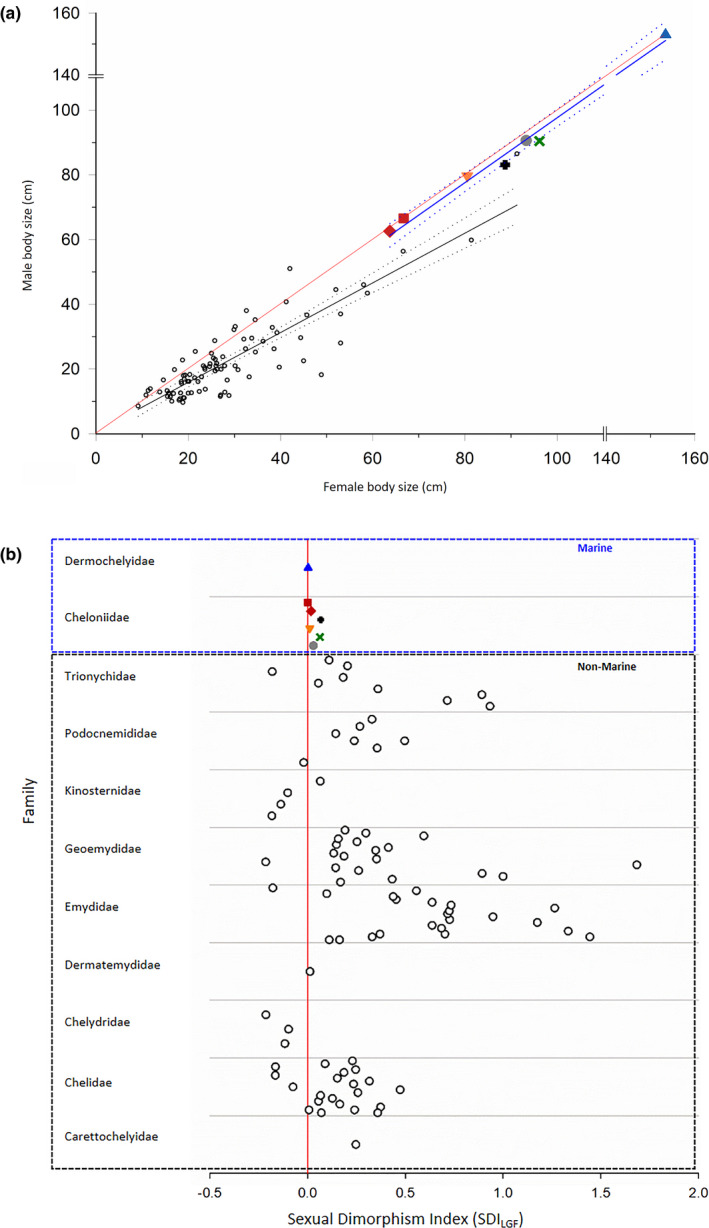
Comparisons of SSD in marine (symbols as in Figure 1) and non‐marine (black open circles) aquatic turtles. (a) Plot of female to male carapace length of marine versus nonmarine aquatic turtles. The red line is the 1:1 line, representing the null hypothesis of no SSD. The solid lines represent the linear regressions and the dotted lines show the 95% confidence intervals. The regression for the marine turtles (blue) is: male CL = −2.4647 + 1.0014 (female CL) adj *r* ^2^ = .99, *p *< .0001). The regression for the NMATs (black) is: male CL = 0.5345 + 0.7684 (female CL) adj *r* ^2^ = .78, *p *< .0001). See text and Table 1a for statistical details. (b) Comparison of Sexual Dimorphism Index (SDI_LGF_) values for marine versus nonmarine aquatic turtles (symbols as Figures 1 and 2a). The vertical red line represents the null hypothesis of no sexual dimorphism (SDI_LGF_ = 0). See text and Table 1b for statistical details. Proportional representation of data among the different turtle families: Chelidae: 21/67 (31%), Carettochelyidae: 1/1 (100%), Cheloniidae: 6/6 (100%), Chelydridae: 3/5 (60%), Dermatemydidae: 1/1 (100%), Dermochelyidae: 1/1 (100%), Emydidae: 22/91 (24%), Geoemydidae: 19/96 (20%), Kinosternidae: 4/41 (10%), Podocnemididae: 7/8 (88%), Trionychidae: 9/45 (20%) (Turtle Taxonomy Working Group et al., 2021)

Availability of body sizes for both sexes within a single population varied widely among the seven species of marine turtles (Table S2). By far, the most data were available for the green turtle (*Chelonia mydas*), which yielded credible estimates from 18 studies of 17 populations (Figure 1a). We found data for six populations for the loggerhead (*Caretta caretta*), four populations for the olive ridley (*Lepidochelys olivacea*), three populations for the hawksbill (*Eretmochelys imbricata*), two populations for the leatherback (*Dermochelys coriacea*), and two studies from the only population of the Kemp's ridley (*Lepidochelys kempii*), and a single population of the flatback (*Natator depressus*). The sex‐specific means and sample sizes are shown in Figure 1b and summarized in Table S2. For the species for which we have estimates from five or more populations (*C*. *mydas*, *C*.* caretta*), it is noteworthy that the degree and direction of SSD vary (Figures 1a,b and 2b).

Figure 1a also illustrates the regression of sex‐specific mean values (male vs. female) for all populations. Neither the slope nor the intercept of this regression differed significantly from the null expectation (slope: *t*(33) = 0.052, *t*_crit_ = 2.034, *p* = 1; intercept: *t*(33) = −0.514, *t*_crit_ = 2.034, *p *= .611). The upper 95% confidence interval of the regression is slightly below the 1:1 line, indicating a weak female‐biased SSD. Examination of the scatter suggests that this overall pattern is driven largely by *C*. *mydas*.

### 3.2 Comparisons of SSD in marine with NMAT

#### 3.2.1 Regression approach

Figure 2 illustrates separate regressions of male versus female body size for marine and NMAT with 95% confidence intervals and the 1:1 line (null hypothesis) for comparison. The regression for marine turtles using only the species‐mean data (Figure 2a) was: male CL = −2.4647 + 1.0014 (female CL) adj *r* ^2^ = .99, *p *< .0001). Neither the slope nor the intercept differed significantly from the null expectation (slope: *t*(4) = −0.001, *t*_crit_ = 2.267, *p *= .99; intercept: *t*(4) = −1.874, *t*_crit_ = 2.267, *p* = 1). The 95% confidence intervals of this regression overlap or encompasses the 1:1 line.

By contrast, NMATs exhibit a wide range of departures from equality in size, mainly in the direction of larger female size (Figure 2a), which is reflected by a slope of 0.76 and 0.82 for the species mean and family mean regression line, respectively (Figure 2a, also see Figure S1 and R Code in Supporting Information). The 95% confidence intervals overlap the 1:1 line only in the domain of the very smallest species (<~15 cm CL). The ANOVA (Table 1a) indicated not only an overall significant effect of female size on male size, but also a significant interaction between female size and habitat, indicating that this effect differed between marine and nonmarine habitats. In other words, in conjunction with the results of the regressions, this means that females are on average larger than males in NMAT, but sexes do not differ in size in marine turtles.

**TABLE 1:** Analyses of Variance (ANOVAs) modeling the degree of difference in body size between males and females in nonmarine versus marine turtles. (a) Analysis of mean body size between males and females (b) Analysis of SDI_LGF_ values

Source of variation	*df*	Type III SS	*F*‐ratio	prob > F
(a) Mean body size				
MODEL‐ adjusted *R* ^2^ = .932099	3	45163.097	426.5480	<.0001
Effects				
Female carapace length	1	13054.492	369.8839	<.0001
Habitat type	1	29.788	0.8440	.3607
Habitat type × female carapace length	1	226.311	6.4123	.0131
Error	90	3176.414		
Corrected total	93	48339.511		
(b) SDI_LGF_				
MODEL‐ adjusted *R* ^2^ = .034656	1	0.6204	4.3387	.04
Effects				
Habitat type	1	0.6204	4.3387	.04
Error	92	13.1547		
corrected total	93	13.7751		

#### 3.2.2 SDI_LG_ approach

Figure 2b illustrates SDI_LG_ values for all 94 species of aquatic turtles (87 nonmarine, seven marine). The values for marine turtles are very close to or overlap the null expectation of no SSD (SDI_LGF_ = 0). The mean SDI_LG_ for marine turtles was 0.027 ± 0.0103 and ranged from 0.002 to 0.067, whereas the mean SDI_LGF_ for NMAT was an order of magnitude higher and biased towards females (0.337 ± 0.0419, range −0.215 to 1.684). ANOVA (Table 1b) indicated that these distributions were significantly different (*F*_1,92_ = 4.339, *p *= .04). Furthermore, the range of values was considerably larger in nonmarine turtles (NMATs) (1.899) than in marine species (0.066).

## 4 DISCUSSION

Our literature review and resulting dataset permitted the first comprehensive statistical evaluation of the degree of SSD in marine turtles, reflecting information from all seven extant species. Although there appears to be weak female‐biased SSD (Figure 1a), primarily driven by *C*. *mydas*, there was no statistically significant difference in SSD when considering data for all species taken together. These results are also reflected in the phylogenetic analysis of SSD in turtles by Gosnell et al. (2009), who documented a nonbiased SSD for the Cheloniidae. This pattern is distinct from NMAT, which typically exhibit female‐ or male‐biased SSD (Figure 2). In the absence of male combat, female‐biased SSD is commonly interpreted as a response to fecundity selection. By contrast, male combat should result in larger male size via sexual selection (Berry & Shine, 1980). Marine turtles do not exhibit male combat in the strict sense of actual physical fighting and, therefore, would be expected to exhibit female‐biased SSD, with all other conditions remaining the same. Whatever the sources of selection, differences between sexes in body size arise due to differences in growth rates over the same prematuration interval or due to differences in the prematuration duration of growth (Bernardo, 1993; Cox & John‐Alder, 2007; Lovich et al., 2014; Stamps, 2008; Stamps & Krishnan, 1997). A third possibility is a differential, size‐specific mortality that influences one sex more than the other (DeGregorio et al., 2012; Roosenburg, 1991). The virtual lack of SSD in marine turtles thus requires an understanding of what kind of selection prohibits differentiation in size between sexes in marine turtles compared to NMAT. We are discussing potential drivers using a morphology‐performance‐fitness argument *sensu* Darwin (1859) or Arnold (1983) and are suggesting that this non‐bias pattern of SSD in marine turtles is due to both the high energetic cost of locomotion in the marine environment coupled with frequent long‐distance movements by both sexes.

It is well‐established that the marine environment imposes strong selection on body form of large, widely‐foraging vertebrates (Fish, 1993, 1998; Fish et al., 2008; Kelley & Pyenson, 2015; Seibel & Drazen, 2007; Webb, 1988; Webb & De Buffrénil, 1990; Williams Terrie, 1999), driven principally by selection for drag reduction and therefore cost‐efficient swimming (Fish, 1993, 1998; Webb, 1988; Williams Terrie, 1999). Saltwater is denser and has higher dynamic and kinematic viscosities than freshwater (Vogel, 1994). Consequently, we would expect from first principles that the marine environment establishes a different selective milieu on sexual dimorphism than the freshwater environment.

Marine turtles are morphologically distinctive among all aquatic turtles in several ways. First, marine turtle limbs reflect a strong selection for specialized locomotion, both in form and function. While all limbs are modified into flippers, the forelimbs are hypertrophied and modified into broad, distally tapered, rigid, wing‐like flippers (Davenport et al., 1984; Renous et al., 2007; Wyneken, 1997). While the NMAT *Carettochelys insculpta* also has wing‐like flippers, the underlying anatomy is still very distinct from marine turtles (Rivera et al., 2013). Apart from the external wing‐like shape, the underlying bony architecture is distinct in marine turtles (Renous et al., 2007). In particular, the humerus is flattened compared to other turtles (Renous et al., 2007; Rivera et al., 2013; Wyneken, 1997) and biomechanical analyses indicate that this confers great strength and hydrodynamic efficiency (Dickson & Pierce, 2019). The locomotory pattern of marine turtles consists of a synchronous upward/downward sweeping motion of the fore flippers that generates thrust (Davenport et al., 1984; Wyneken, 1997), similar to the pattern in other marine tetrapods that have flippers (Clark & Bemis, 1979; Walker, 2002). That such derived flippers have convergently evolved across multiple lineages of other marine tetrapods, including seals, penguins, and plesiosaurs (Wyneken, 1997), indicates strong selection for efficient long‐distance swimming. It is well established from mathematical modeling that flapping appendages in large aquatic animals permit efficient and rapid propulsion (Blake, 1981; Walker, 2002; Walker & Westneat, 2000).

The second morphological specialization of marine turtles is the extraordinary streamlining of their body form compared to most other fully aquatic turtles (Davenport et al., 1984; Wyneken, 1997) within the limitations of the turtle Bauplan which is defined by a heavy, bony shell (Gilbert et al., 2007; Wyneken, 2001, 2003). The stereotypic streamlining is evident throughout their evolutionary history, including the oldest known definitive species, *Desmatochelys padillai* (Cadena & Parham, 2015), and across an order of magnitude range in body size from the smallest living species *Lepidochelys kempii* (~63 cm carapace length) to the largest known species, the extinct *Archelon ischyros* (Wieland, 1896), which exceeded 400 cm in carapace length. The limitation of streamlining imposed by the shell is further suggested by the fact that the most streamlined marine turtles (such as *A*. *ischyros* and *D*. *coriacea*) have secondarily reduced or lost the bony structures of the shell (Bang et al., 2016; Gilbert et al., 2007; Wieland, 1896; Wyneken, 2001; Zangerl, 1980). A further adaptation evident in *D*. *coriacea* are the longitudinal dorsal ridges on the carapace that enhance hydrodynamic performance (Bang et al., 2016; Davenport et al., 2011).

These specializations taken together indicate that selection has optimized marine turtle morphology for energetically efficient swimming. Indeed, a key feature of marine turtle biology is their capacity to exploit resources across vast geographic expanses. Both sexes of all seven species of marine turtles undertake long‐distance, sometimes trans‐oceanic migrations covering many hundreds to thousands of kilometers (Boyle et al., 2009; Hays et al., 2004, 2014; Hays & Scott, 2013; Luschi et al., 2003; Plotkin, 2003, 2010; Shillinger et al., 2008). Two species (*D*. *coriacea* and *L*. *olivacea*) are oceanic, pelagic, and widely‐foraging predators (Hays et al., 2004; Plotkin, 2010; Shillinger et al., 2008). Satellite tagging studies show that *D*. *coriacea* achieves a mean speed of 33–49 km/day, with a maximum of 62 km/day (Shillinger et al., 2008) and *L*. *olivacea* with a mean of 28.32 km/day and a maximum of 79.4 km/day (Plotkin, 2010). Future studies might want to consider this argument in the context of large, mobile marine vertebrates but also consider mating systems.

Despite their having evolved unique morphology among turtles for efficient swimming, this high vagility lifestyle also entails substantial energetic expenditure. Unfortunately, few quantitative data on the energetic requirements of swimming in adult marine turtles are available. However, Prange (1976) studied the metabolic cost of swimming in juvenile *Chelonia mydas*. By extrapolating these costs, he estimated that the energy demand for long‐distance migration of adults between breeding and feeding grounds would require approximately 21% of their body mass in fat stores. Given the common body form and long‐distance movement of all marine turtle species, it is not far‐fetched that all species incur these energy requirements.

An alternative hypothesis for the minimal SSD observed in marine turtles might be the high energetic costs of sustained swimming incurred by both sexes. Neither sex can allocate significant energy to continued growth after maturation, and therefore neither sex can achieve a larger size than the other (Bernardo, 1993; Cox & John‐Alder, 2007; Stamps, 2008; Stamps & Krishnan, 1997). For instance, female‐biased SSD is usually attributed to fecundity selection. However, the ability of female marine turtles to allocate energy to enhanced postmaturation growth in response to fecundity selection appears to be prohibited by their costly migration. While males may not incur high costs for gamete production, unlike females that skip nesting seasons, males likely incur higher energetic costs due to their annual migrations to mating and nesting grounds (Hatase & Tsukamoto, 2008; Hays et al., 2010).

A further indication that marine turtles lack discretionary energy for continued growth that could produce SSD is found in their unusual postmaturation growth patterns. Marine turtles exhibit determinate‐like growth (Omeyer et al., 2018), a pattern which is unlike other turtles (Congdon et al., 2013; Gibbons et al., 1981; Lindeman, 1999) and in fact unlike most other ectotherms (Bernardo, 1993; Gotthard, 2001; Sebens, 1987; Tilley, 1980) which exhibit indeterminate growth.

In conclusion, our study demonstrated that, unlike NMAT, marine turtles are only minimally sexually size dimorphic. We argued that this difference is due to the distinct selective milieu imposed by the oceanic environment. Hence, future studies should acknowledge this distinction and no longer group marine turtles with NMAT as numerous studies have previously done and account for the differences in habitat that may affect SSD. However, comparative studies, including ours, are limited by the data available. Many species only have data for a very limited amount of populations available. Our data reflect what Lovich et al. (2010) already previously observed. The magnitude and direction of SSD can vary depending which population of a species is studied. This indicates that the common practice of basing a species‐level trait estimate on a single population likely introduces error variance in comparative datasets in general. Therefore, where data are available for multiple populations of the same species (Figure 1a), it should be included in analyses of SSD. Further, we note that in our comprehensive dataset, the sample size for females was more than 20 times that for males, and in the case of *N*. *depressus*, only a single male was measured. This unbalanced sample size between sexes is likely due to the oversampling of nesting females; future studies need to be deliberate about acquiring male data on marine turtle males.

## AUTHOR CONTRIBUTIONS

**Christine Figgener:** Conceptualization (equal); Data curation (lead); Formal analysis (equal); Investigation (lead); Methodology (equal); Project administration (lead); Validation (equal); Visualization (lead); Writing – original draft (equal); Writing – review & editing (lead). **Joseph Bernardo:** Conceptualization (equal); Formal analysis (equal); Investigation (supporting); Methodology (equal); Validation (equal); Visualization (supporting); Writing – original draft (equal); Writing – review & editing (supporting). **Pamela Plotkin:** Funding acquisition (lead); Visualization (supporting); Writing – original draft (supporting); Writing – review & editing (supporting).

## CONFLICT OF INTEREST

The authors declare no competing interests.

### OPEN RESEARCH BADGES

This article has been awarded Open Data and Open Materials Badges. All materials and data are publicly accessible via the Open Science Framework at: https://doi.org/10.5061/dryad.v6wwpzgzg.

## Supporting information

Table S1‐S4

Supplementary Material

## Data Availability

The datasets supporting this article are available in Dryad https://doi.org/10.5061/dryad.v6wwpzgzg.
