# Supplementary material for: Marine turtles are only minimally sexually size dimorphic, a pattern that is distinct from most nonmarine aquatic turtles
Source: Ecol Evol. 2022 Jun 2;12(6):e8963. doi: 10.1002/ece3.8963 (PMC9163671; doi:10.1002/ece3.8963)
Supplement: Supplementary file 2 — Supplementary Material [file ECE3-12-e8963-s001.docx]

**Supplementary Materials**

Contents:

**Supplementary Methods**

**Column Descriptions Supplementary Table S1. A summary of data from the primary literature reported in six prior reviews and their respective supplementary datasets that we examined for accuracy and completeness.**

**Column Descriptions Supplementary Table S2. A new compilation of mean body size data for 36 different populations of the seven species of marine turtles based on examination of primary sources.**

**Column Descriptions Supplementary Table S3. Compilation of mean body size data of 94 species of marine and non-marine but fully aquatic turtles.**

**Column Descriptions Supplementary Table S4. Mean body size data of 11 turtle families containing only the 94 marine and non-marine but fully aquatic species.**

**Supplementary Table S5: Proportional representation of data IN OUR ASSEMBLED DaTASET among the DIFFERENT TURTLE families**

**Supplementary Figure S1. Regression of NMAT family mean body size data and comparison to the 1:1 line (intercept 0, slope 1), indicating no differences in body size between males and females.**

**R Code for the comparison of a regression line to the 1:1 line (intercept 0, slope 1)**

**Citations of review studies**

**Supplementary Methods**

*Methods for Compiling a Novel, Comprehensive Dataset of Marine Turtle Body Size.*

To assemble a dataset specifically for marine turtle body size, we started by examining the marine turtle data used in seven comprehensive reviews and analyses aimed at understanding patterns of sexual size dimorphism in turtles [1-7]. A cursory examination of the datasets used in these papers indicated that there were inaccuracy and omissions and that many datasets simply copied previous data compilations, even if subsequent studies included new data. For instance, none of the previous reviews included data for all seven species of marine turtles never, often only including values for a single population and representative of a species. Consequently, we undertook a comprehensive examination of the primary literature to improve existing datasets. To do this, we examined each primary literature source cited in the prior reviews, and we quality checked each data point. A summary of all the primary literature we examined and how it relates to the accuracy and completeness of the data reported in the prior reviews is detailed in Supplementary Table S1.

Next, we conducted a literature search using Google Scholar, SCOPUS and the literature database on [SeaTurtle.org](http://www.seaturtle.org/library/) to identify any additional primary literature reporting body size data for marine turtles. We included peer-reviewed studies, student theses, and reports that reported body size data for both sexes within a species and population. We only accepted values when it was clear that they were based on sexually mature adults. We tabulated carapace length means, standard error (SE), and sample sizes. We accepted means from studies of any species that reported the origin of samples, sample size, and either SD or SE within one nesting population or foraging area. We also accepted values from studies for which we could compute means and SE from either original supplementary datasets if available, or from datasets we generated by extracting values from published figures using PlotDigitizer 2.6.88. If multiple estimates (different populations) existed for the same species, we accepted all of them. The resulting dataset (Supplementary Table S2) included credible estimates from 36 different populations comprising all seven species of marine turtles with most represented by more than one population.

*Comprehensive Compilation of Body Size Data of Marine and Non-marine Turtles.*

We generated a dataset for non-marine aquatic turtle body size data by combining the information from the two reviews that included data for the largest number of turtle species and because they specifically coded the aquatic species [1, 4]. Due to taxonomic changes, sometimes data appeared to come from two different species when in fact there were two names that applied to the same species (e.g. an older name and a newer name). Therefore, we reconciled the species’ names using the Annotated Checklist “Turtle Species of the World” [8]. We did not include any of the data for marine turtles from these papers but rather added our new estimates of species mean values that we calculated from the data in Supplementary Table 2. We coded each species as marine (M) or non-marine aquatic (A). Our final turtle body size database contains data for 94 species (seven marine and 87 non-marine aquatic) and is available in Supplementary Table S3 with mean size data per species and in Supplementary Table S4 with mean body size data per turtle family.

**Column Descriptions Supplementary Table S1. A summary of data from the primary literature reported in six prior reviews and their respective supplementary datasets that we examined for accuracy and completeness.**

Confirmed Were we able to confirm used data from cited primary source (Yes/No)

Citation Citation of primary source

Species Marine turtle species: Cc = *Caretta caretta*, Cm = *Chelonia mydas*, Dc = *Dermochelys coriacea*, Ei = *Eretmochelys imbricata*, Lk = *Lepidochelys kempii*, Lo= *Lepidochelys olivacea*, Nd = *Natator depressus*

Location Location of sample collection

Included in Study Citation of study that included values of given primary source

n.F Sample size of females

F.CL Female carapace length mean value

n.M Sample size of males

M.CL Male carapace length mean value

**Column Descriptions Supplementary Table S2. A new compilation of mean body size data for 36 different populations of the seven species of marine turtles based on examination of primary sources.**

Citation Primary source of data

Species Marine turtle species: Cc = *Caretta caretta*, Cm = *Chelonia mydas*, Dc = *Dermochelys coriacea*, Ei = *Eretmochelys imbricata*, Lk = *Lepidochelys kempii*, Lo= *Lepidochelys olivacea*, Nd = *Natator depressus*

Location Location where samples were taken

Basin Ocean basin the samples were taken

CL What type of measurment of carpace length were taken (CCL = Curved Carapace Length, SCL = Straight Carapace Length)

Female.N Female sample size

Female.CL Female carapace length mean value

Female.CL.Range Range of values

Female.CL.SE Standard Error

Male.N Male sample size

Male.CL Male carapace length mean value

Male.CL.Range Range of values

Male.CL.SE Standard Error

**Column Descriptions Supplementary Table S3. Compilation of mean body size data of 94 species of marine and non-marine but fully aquatic turtles.**

Species Turtle species

Family Turtle family

n (populations) Number of populations surveyed and included in mean values

Larger sex The larger sex in this species (M = males, F = Females)

F.CL Female carapace length mean value

F.CL.SE Standard error of mean carapace length of females

M.CL Male carapace length mean value

M.CL.SE Standard error of mean carapace length of males

Habitat_Type Main habitat for species (A = fully aquatic, M = marine)

Habitat Main habitat for species coded (1 = fully aquatic, 2 = marine)

SDI_ln Sexual Dimorphism Index based on Logarithm

SDI_LG Sexual Dimorphism Index based on Formula by Lovich-Gibbons (1992) and limited to one equation as suggested by Fairbairn (1997)

**Column Descriptions Supplementary Table S4. Mean body size data of 11 turtle families containing only the 94 marine and non-marine but fully aquatic species.**

Family Turtle family

n (species) Number of species in family

Larger sex Larger sex in turtle family

Habitat_Type Preferred habitat of turtle family (A = fully aquatic, M = marine)

Habitat Preferred habitat of turtle family coded (1 = fully aquatic, 2 = marine)

F.CL Female carapace length mean value

M.CL Male carapace length mean value

SDI_ln Sexual Dimorphism Index based on Logarithm

SDI_LG Sexual Dimorphism Index based on Formula by Lovich-Gibbons (1992) and limited to one equation as suggested by Fairbairn (1997)

**SUPPLEMeNTARY TABLE S5: Proportional representation of data IN OUR ASSEMBLED DaTASET among the DIFFERENT TURTLE families*:**

Chelidae: 21/67; (31%)

Carettochelyidae: 1/1; (100%)

Cheloniidae: 6/6; (100%)

Chelydridae: 3/5; (60%)

Dermatemydidae: 1/1; (100%)

Dermochelyidae: 1/1; (100%)

Emydidae: 22/91; (24%)

Geoemydidae: 19/96; (20%)

Kinosternidae: 4/41; (10%)

Podocnemididae: 7/8; (88%)

Trionychidae: 9/45; (20%).

*Based on [8]

**Supplementary Figure S1. Regression Line (black) of NMAT family mean body size data and comparison to the 1:1 line (intercept 0, slope 1) (red), indicating the NuLL hypothesis of no differences in body size between males and females.**

**
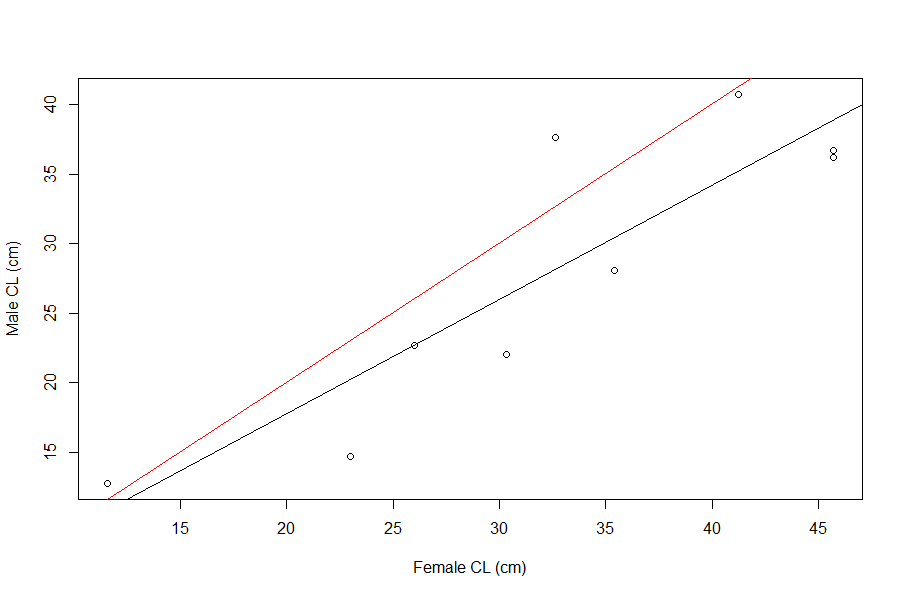
**

**R Code for the comparison of a regression line to the 1:1 line (intercept 0, slope 1)**

turt.fam.sd <- read.csv("Data Mining_Non-Marine Turtle SD_FamilyMeans_DataR.csv", header = TRUE, sep = ",", dec = ".", na.strings=c("NA", "NULL", ""))

head(turt.fam.sd)

str(turt.fam.sd)

plot(turt.fam.sd$F.CL, turt.fam.sd$M.CL,main=NA,xlab="Female CL (cm)",ylab="Male CL (cm)")

*#H_Null: slope=0; H_alternative: slope!=0*

Turt.model.4 <- lm(M.CL~F.CL, data=turt.fam.sd)

turt.model.4

*#Coefficients:*

*# (Intercept) F.CL*

*# 1.3422 0.8208 (=slope)*

abline(coef(turt.model.4))

sum.4 <- summary(turt.model.4)

str(sum.4)

*#Calculate S.E. of regression*

sum.4$sigma

*#5.216016*

5.216016/(sqrt(9)) # 9 = Sample size

*#SE=1.738672*

*#Analysis of Variance Table for Model*

anova(turt.model.4)

*#Response: M.CL*

*# Df Sum Sq Mean Sq F value Pr(>F)*

*# F.CL 1 680.01 680.01 24.994 0.001566 ***

*#Residuals 7 190.45 27.21*

*#---*

*# Signif. codes: 0 '***' 0.001 '**' 0.01 '*' 0.05 '.' 0.1 ' ' 1*

###############################################################################

*#H_Null: slope=1; H_alternative: slope!=1*

*#Values from turt.model.4 and calculated SE above*

*#sample size=9; intercept= 1.3422 ; slope=0.8208; SE=1.738672*

*# Testing the contrasts*

*# beta_0 = 0 and*

*# beta_1 = 1*

*# However, we can't use multcomp to test the contrasts because (i) multcomp*

*# doesn't let you work with the intercept and (ii) it tests contrasts separately,*

*# You need a global test of both contrasts at once.*

*#Extra steps included below (like subtracting 0) to explain formula*

turt.model.4 <- lm(M.CL~F.CL, data=turt.fam.sd)

*# in the plot below, the red line is the intercept 0, slope 1 ( y = x) line and the black line is the regression line for the NMAT family mean body size data.*

plot(turt.fam.sd$F.CL, turt.fam.sd$M.CL, main=NA, xlab="Female CL (cm)",ylab="Male CL (cm)")

abline(0,1, col="red"); abline(turt.model.4)

summary(turt.model.4)

*# Estimate Std. Error t value Pr(>|t|)*

*#(Intercept) 1.3422 5.5959 0.240 0.81731*

*#F.CL 0.8208 0.1642 4.999 0.00157 ***

anova(turt.model.4)

*# Df Sum Sq Mean Sq F value Pr(>F)*

*#Female.CL 1 680.01 680.01 24.994 0.001566 ***

*#Residuals 7 190.45 27.21*

*# Simultaneously test H0: (beta_0, beta_1) = (0, 1)*

v <- c(1.3422, 0.8208) - c(0, 1) # v <- c(intercept, slope) –c (0,1) = difference between lm values and H0 values

X <- model.matrix(turt.model.4)

*# In the calculation below:*

*# * you divide by 2 is because you're testing two contrasts*

*# * you divide by sample size - number of parameters in your model*

*# = 7 - 2 for this example.*

*#If you had a multiple regression mode*

*# like y ~ x1 + x2 + x3, you'd subtract 4, even if you were only testing*

*# two contrasts.*

*# * All other numbers come from the summary or anova above*

F_0 <- t(v) %*% t(X) %*% X %*% v / 2 / (27.2/(7)) #mean square of Residuals from anova/DF (n-parameters)

(p.value <- pf(F_0, 2, 7, lower.tail=F))

*# 0.0004980228 = p-value*

*# the slope of the regression line of NMAT family mean body size values is significantly smaller #(p<0.05) than 1, which means the regression line is significantly different*

*#from the Intercept 0, Slope 1-Line*

**Citations**

[1] Agha, M., Ennen, J.R., Nowakowski, A.J., Lovich, J.E., Sweat, S.C. & Todd, B.D. 2018 Macroecological patterns of sexual size dimorphism in turtles of the world. *Journal of Evolutionary Biology* **31**, 336-345. (doi:10.1111/jeb.13223).

[2] Ceballos, C.P., Adams, D.C., Iverson, J.B. & Valenzuela, N. 2013 Phylogenetic patterns of sexual size dimorphism in turtles and their implications for Rensch’s rule. *Evolutionary Biology* **40**, 194-208. (doi:10.1007/s11692-012-9199-y).

[3] Halámková, L., Schulte, I.I.J.A. & Langen, T.A. 2013 Patterns of sexual size dimorphism in *Chelonia*. *Biological Journal of the Linnean Society* **108**, 396-413. (doi:10.1111/j.1095-8312.2012.02015.x).

[4] Regis, K.W. & Meik, J.M. 2017 Allometry of sexual size dimorphism in turtles: a comparison of mass and length data. *PeerJ* **5**, e2914. (doi:10.7717/peerj.2914).

[5] Gibbons, J.W. & Lovich, J.E. 1990 Sexual dimorphism in turtles with emphasis on the slider turtle (*Trachemys scripta*). *Herpetological Monographs* **4**, 1-29.

[6] Berry, J.F. & Shine, R. 1980 Sexual size dimorphism and sexual selection in turtles (Order Testudines). *Oecologia* **44**, 185-191.

[7] Gosnell, S.J., Rivera, G. & Blob, R.W. 2009 A phylogenetic analysis of sexual size dimorphism in turtles. *Herpetologica* **65**, 70-81. (doi:10.1655/07-057r2.1).

[8] Turtle Taxonomy Working Group, Rhodin, A.G.J., Iverson, J.B., Bour, R., Fritz, U., Georges, A., Shaffer, H.B. & van Dijk, P.P. 2017 Turtles of the World: Annotated Checklist and Atlas of Taxonomy, Synonymy, Distribution, and Conservation Status. In *Conservation Biology of Freshwater Turtles and Tortoises: A Compilation Project of the IUCN/SSC Tortoise and Freshwater Turtle Specialist Group* (eds. A.G.J. Rhodin, J.B. Iverson, P.P. van Dijk, R.A. Saumure, K.A. Buhlmann, P.C.H. Pritchard & R.A. Mittermeier), pp. 1-292, 8th ed, Chelonian Research Monographs.
